# Perceived academic support and academic achievement among Ethiopian first-year university students: the serial mediating roles of academic motivation and resilience

**DOI:** 10.3389/fpsyg.2025.1605550

**Published:** 2025-07-22

**Authors:** Biniyam Kerebeh Melaku, Reda Darge Negasi, Tiruwork Tamiru Tolla

**Affiliations:** Department of Psychology, Bahir Dar University, Bahir Dar, Ethiopia

**Keywords:** academic achievement, academic motivation, academic resilience, perceived academic support, serial mediation model

## Abstract

**Introduction:**

This study investigates the mediating roles of academic motivation and resilience in the relationship between perceived academic support and academic achievement among Ethiopian first-year university students, addressing a gap in understanding how these protective factors enhance academic achievement during a critical transitional period.

**Methods:**

A correlational design was employed, collecting data from first-year students at Injibara University. Instruments included the Perceived Academic Support Scale (Gutiérrez et al., validated by Reyes et al.), a revised 20-item Academic Resilience Scale (Cassidy, adapted by Cui, Wang, & Xu for collectivist contexts), and the Multidimensional Academic Motivation Scale (Vallerand et al., adapted by Orsini for university settings). Participants were selected via stratified random sampling, stratified by gender and college affiliation. Data collection occurred in two phases: a pilot study (*n* = 200; 158 male, 42 female) validated the instruments, followed by the main study (*n* = 503; 403 male, 100 female). Pearson correlation coefficients was used to analyze correlation among the variables. Besides structural equation modeling (SEM) was used to examine the direct and indirect effect of perceived academic support on achievement through the mediators of academic motivation and resilience.

**Results:**

Pearson correlations revealed significant positive associations among perceived academic support, academic motivation, academic resilience, and academic achievement (*p* < 0.05). Analysis from the structural equation modeling confirmed that academic motivation and resilience fully mediated the relationship between perceived academic support and academic achievement, with robust model fit indices.

**Discussion:**

These findings highlight the critical roles of motivation and resilience as mechanisms linking support to achievement, offering practical implications for educators to bolster student success in the first year. Limitations include the study's focus on a cross-sectional design and a single institution. Future research should explore longitudinal designs and diverse cultural contexts to enhance generalizability.

## Introduction

The transition to university is a critical phase for first-year students, marked by academic, social, and emotional challenges that shape their educational trajectories. Global research underscores the importance of adaptive mechanisms for academic success, grounded in theoretical frameworks like Self-Determination Theory (SDT) of motivation, the Stress and Coping Theory's buffering model, and Cassidy's academic resilience model (Ryan and Deci, 2000; Cohen and Wills, 1985; Cassidy, 2016; Lakey and Cohen, 2000). SDT posits that intrinsic and extrinsic motivation, driven by autonomy, competence, and relatedness, enhances engagement and achievement (Ryan and Deci, 2000). However, stressors—separation from family, academic demands, and social maladjustment—threaten wellbeing and increase dropout risks (Chemers et al., 2001; Robotham, 2008). In Ethiopia's rapidly expanding higher education system, these challenges are pronounced. Kelemu and Sabanci (2018) report elevated dismissal rates, with Tamrat (2021) documenting a 36% attrition rate across seven public universities, peaking during the first-to-second-year transition. Audits at Hawassa and Dambi Dollo Universities reveal dropout rates of over 30% and 14%, respectively (Tamrat, 2021; Alemu et al., 2022), undermining institutional and national development goals.

Stress and Coping Theory's buffering model suggests that perceived academic support from peers, family, and instructors mitigates stress's adverse effects, fostering psychological wellbeing and achievement (Cohen and Wills, 1985; Awang et al., 2014). Academic support is defined as tangible and emotional assistance enhancing students' coping resources. *Similarly*, Cassidy's (2016) academic resilience model emphasizes adaptive responses—perseverance, reflecting, and help-seeking—that enable students to overcome academic adversity. Motivation, as per SDT, and resilience collectively buffer stressors, enhancing performance (Vallerand et al., 1992; Cassidy, 2016). Yet, Ethiopia-specific research on these constructs is limited. Studies like Niguse et al. (2019), Mekonnen (2018), and Mekonnen (2021) explore social support but fail to examine its mediating effects on first-year achievement through motivation and resilience. Debele (2020) and Amare (2014) analyze motivation using basic correlations, overlooking SDT-driven mediation pathways. This study integrates Self-Determination Theory (SDT), Stress and Coping Theory, and Cassidy's resilience model to provide a comprehensive framework for understanding how academic support influences achievement through academic motivation and resilience among first-year university students. By combining SDT's focus on intrinsic and extrinsic motivation, Stress and Coping Theory's emphasis on managing academic stressors, and Cassidy's resilience model's insights into adaptive coping strategies, the study addresses global challenges in educational psychology, such as enhancing student motivation and improving learning outcomes across diverse populations. Globally, this integrated model offers a scalable theoretical lens to design interventions that foster motivation and resilience in varied educational systems, accommodating cultural and socioeconomic differences. For instance, SDT's universal principles of autonomy, competence, and relatedness can guide global policies on student-centered learning, while Stress and Coping Theory and Cassidy's model inform strategies to mitigate universal stressors like academic transitions.

Context-specifically, the model provides evidence-based insights tailored to first-year students at Injibara University, Ethiopia, where resource constraints and cultural factors shape academic experiences. The findings highlight the role of targeted academic support (e.g., mentorship, peer programs) in boosting motivation and resilience in this under-resourced setting, addressing local challenges like high dropout rates and limited institutional support. By linking global theoretical constructs to context-specific interventions, the study contributes to the educational psychology literature with a versatile framework that informs both universal and localized practices, paving the way for future research to test its applicability across different cultural and educational contexts.

### Conceptual framework

**Figure 1:**
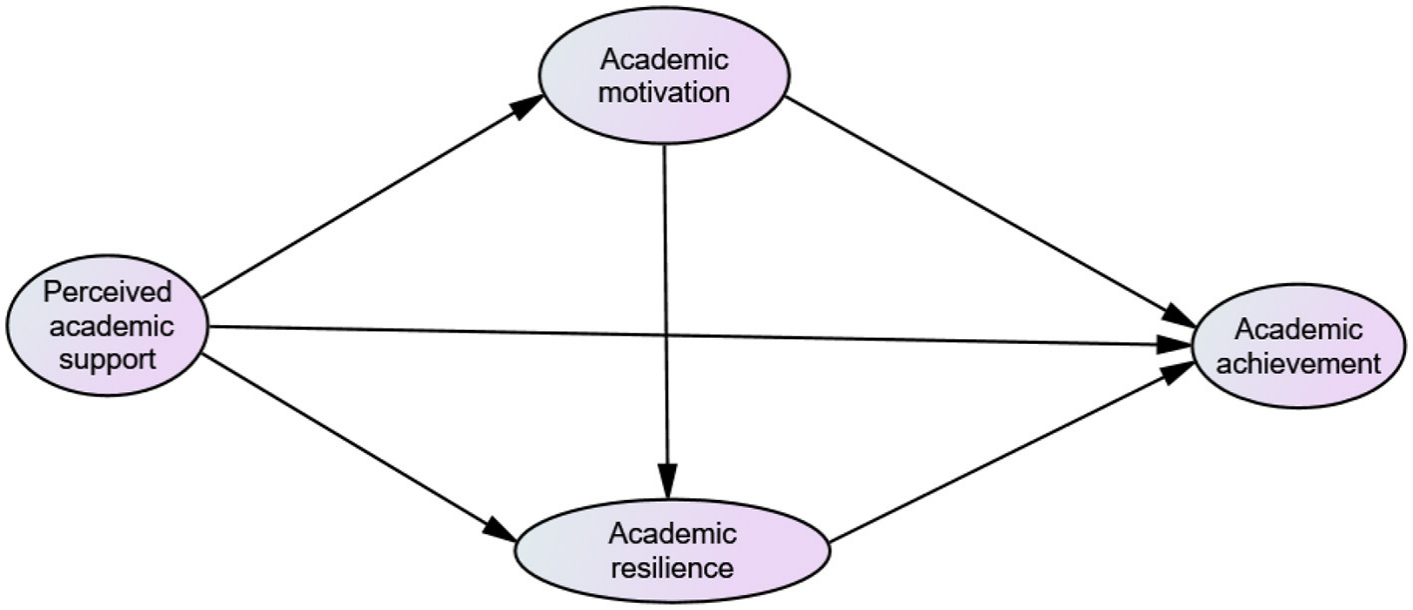
Hypothesized conceptual framework of the study.

This study examines how perceived academic support influences academic achievement among first-year Ethiopian university students, with academic motivation and resilience as mediators. Grounded in Stress and Coping Theory-buffering model (Cohen and Wills, 1985), Self-Determination Theory (Deci and Ryan, 1985), and Cassidy's (2016) Academic Resilience model, we propose a conceptual framework that illustrates the interplay among these constructs. As shown in the Figure 1, perceived academic support may promote academic motivation, which enhances academic resilience, ultimately leading to better academic achievement.

The following hypotheses are tested:

H1a: Perceived academic support directly impacts academic achievement.
H1b: Perceived academic support directly impacts academic motivation.
H1c: Perceived academic support directly impacts academic resilience.
H1d: Academic motivation mediates the relationship between perceived academic support and academic achievement.
H1e: Academic resilience mediates the relationship between perceived academic support and academic achievement.
H1f: Academic motivation via academic resilience mediates the relationship between perceived academic support and academic achievement.
H2: Academic motivation directly impacts academic achievement.
H3: Academic resilience directly impacts academic achievement.

This framework and hypotheses, analyzed via structural equation modeling (SEM), address gaps in Ethiopian higher education research, offering insights for supporting first-year success.

## Methods and materials

### Research design

This study utilized a correlational research design to measure associations among perceived academic support, academic motivation, academic resilience, and academic achievement via statistical correlation tests (Creswell, 2014). Data were collected using questionnaires, suitable for addressing quantitative research questions requiring numerical data.

### Study population, sampling techniques, and sample size

#### Population of the study

The target population comprised first-year students at a public university in Ethiopia, selected for their unique challenges—family separation, new teaching methods, social connections, and career decisions—impacting wellbeing, motivation, resilience, and academic achievement (Tinto, 1993; Elnaem et al., 2024).

#### Sampling technique

Participants were purposefully selected using stratified and simple random sampling. The population was stratified by college (social science and natural science streams) and gender, with proportional allocation to ensure representativeness (Kothari, 2004). A random lottery method minimized sampling bias.

#### Sample size

From a total population of 1,014 first-year students (816 males, 198 females), 760 were sampled across pilot and main studies: 210 for the pilot (165 males, 45 females) and 550 for the main study (441 males, 109 females). The pilot sample followed Kline's (2011) guidelines for structural equation modeling (SEM), while the main study adhered to Sim et al.'s (2021) mean sample size for mediation model complexity, adjusted for a 10% non-response rate. The pilot study yielded complete data for 200 participants (158 males, 42 females; 95.2% completion rate), excluding 10 (4.8%) due to incomplete responses. The main study obtained complete data for 503 participants (403 males, 100 females; 91.5% completion rate), excluding 47 (8.5%) for similar reasons.

### Variables of the study

#### Predictor variable

Perceived Academic Support (family, peer, and teacher support).

#### Mediator variables

Academic Motivation (intrinsic motivation to know, toward accomplishment, to experience stimulation; external, identified regulation, introjected regulation, and amotivation) and Academic Resilience (perseverance, self-reflection & adaptation, adaptive help-seeking, negative affect & emotional response).

#### Criterion variable

Academic Achievement (semester GPA).

### Measurement instruments

#### Measures of perceived academic support

The Perceived Academic Support Scale (PASS; Gutiérrez et al., 2018), originally developed to assess support from family, peers, and teachers, was adapted and validated by Reyes et al. (2022). This study further adapted and validated it for the local context. The 12-item PASS includes subscales for family (e.g., “My parents help me perform well academically”), peers (e.g., “In my university, I have a friend who cares about my education”), and teachers (e.g., “In my university, there is a teacher who cares about my education”), rated on a 5-point scale (1 = strongly disagree, 5 = strongly agree). Prior studies (Gutiérrez et al., 2018; Reyes et al., 2022) and this research confirmed its reliability and validity.

#### Measures of academic motivation

The Multidimensional Academic Motivation Scale (AMS-28; Vallerand et al., 1992) was adapted and validated by Orsini (2016) for university settings. This study further adapted it locally to assess students' reasons for pursuing education. The 28-item scale, scored on a 5-point scale (1 = not at all corresponds, 5 = corresponds exactly), includes intrinsic motivation (12 items: to know, accomplishment, stimulation), extrinsic motivation (12 items: external, identified, introjected regulation), and amotivation (4 items). Higher scores indicate stronger motivation types. Reliability and validity were established previously (Vallerand et al., 1992; Orsini, 2016) and confirmed here.

#### Measures of academic resilience

The Academic Resilience Scale (ARS; Cassidy, 2016) was revised from 30 to 20 items by Cui et al. (2023) for collectivist contexts, with original author permission. This study adapted and validated it locally. The 20-item ARS, rated on a 5-point Likert scale (1 = unlikely, 5 = likely), comprises perseverance (6 items, e.g., “I would use the feedback given to improve my work”), self-reflection/adaptation (6 items, e.g., “I would try to think more about my strengths and weaknesses”), adaptive help-seeking (4 items, e.g., “I would seek encouragement from my family”), and negative affect/emotional response (4 items, e.g., “I would feel like everything was ruined”). Prior studies (Cassidy, 2016; Cui et al., 2023) and this research confirmed its psychometric properties.

#### Measures of academic achievement

Academic achievement was operationalized as students' semester grade point average (GPA), obtained from the university registrar's office.

### Validation of instruments

Instruments were validated through Confirmatory Factor Analysis (CFA), reliability assessments, and convergent/discriminant validity evaluations, leveraging established theoretical frameworks (Byrne, 2016). Data were screened for accuracy, ensuring scores ranged from 1 to 5 with no missing values.

#### Confirmatory factor analysis (CFA)

Confirmatory factor analysis validated the instruments' local applicability, omitting exploratory factor analysis due to prior validation (Byrne, 2016). Model fit was assessed using chi-square (χ^2^/df < 3, *p* >0.05), GFI, NFI, CFI (> 0.90), and RMSEA (< 0.08) (Hu and Bentler, 1999; Collier, 2020; Hooper et al., 2008). Based on these criteria, the result of the pilot study are: Perceived academic support scale(χ^2^ = 72.2, df = 51, χ^2^/df = 1.42, GFI = 0.95, NFI = 0.94, CFI = 0.98, RMSEA = 0.046); Academic motivation scale (χ^2^ = 529.7, df = 278, χ^2^/df = 1.9, GFI = 0.924, NFI = 0.912, CFI = 0.975, RMSEA = 0.031); Academic resilience scale (χ^2^ = 215.1, df = 163, χ^2^/df = 1.32, GFI = 0.91, NFI = 0.94, CFI = 0.99, RMSEA = 0.040).

Moreover, the results of the main study are: Perceived academic support scale (χ^2^/df = 2.1, GFI = 1.00, NFI = 1.00, CFI = 1.00, RMSEA = 0.048); Academic motivation scale (χ^2^/df = 1.6, GFI = 0.93, NFI = 0.92, CFI = 0.97, RMSEA = 0.036); Academic resilience Scale (χ^2^/df = 2.0, GFI = 0.94, NFI = 0.94, CFI = 0.97, RMSEA = 0.046). These indices confirm robust fit of the models.

#### Reliability assessment

Reliability was assessed via Cronbach's alpha (>0.70; Nunnally, 1978; Collier, 2020). PASS subscales: family (α = 0.89), peers (α = 0.82), teachers (α = 0.82). AMS subscales: intrinsic to know (α = 0.89), other motivation/regulation (α = 0.77–0.84). ARS subscales: perseverance (α = 0.95), reflection/adaptation (α = 0.93), help-seeking (α = 0.92), affect/emotional response (α = 0.94). All exceeded thresholds, indicating high consistency (see Table 1).

**Table 1:** Reliability and convergent validity assessment.

**Convergent validity**				
**Constructs**	**Items**	**Loading**	**AVE**	**Cronbach's Alpha**
**Perceived academic support**				
Perceived family support	PASq1	0.78	0.75	0.89
	PASq2	0.75		
	PASq3	0.82		
	PASq4	0.84		
	PASq5	0.74		
	PASq6	0.67		
Perceived peer support	PASq7	0.67	0.76	0.82
	PASq8	0.84		
	PASq9	0.82		
Perceived teacher support	PASq10	0.71	0.78	0.82
	PASq11	82		
	PASq12	81		
**Academic motivation**				
Intrinsic motivation to know	AMq1	0.84	0.83	0.89
	AMq2	0.85		
	AMq3	0.80		
	AMq4	0.83		
Intrinsic motivation toward the accomplishment	AMq5	0.78	0.74	0.83
	AMq6	0.79		
	AMq7	0.68		
	AMq8	0.71		
Intrinsic motivation to experience stimulation	AMq9	0.66	0.71	0.79
	AMq10	0.72		
	AMq11	0.77		
	AMq12	0.68		
External regulation	AMq13	0.66	0.71	0.80
	AMq14	0.78		
	AMq15	0.74		
	AMq16	0.76		
Identified regulation	AMq17	0.75	0.70	0.77
	AMq18	0.68		
	AMq19	0.72		
	AMq20	0.69		
Introjected regulation	AMq21	0.69	0.72	0.81
	AMq22	0.75		
	AMq23	0.72		
	AMq24	0.71		
Amotivation	AMq25	0.75	0.75	0.84
	AMq26	0.71		
	AMq27	0.79		
	AMq28	0.75		
	PASq11	0.84		
	PASq12	0.85		
**Academic resilience**				
Perseverance	ARq1	0.92	0.87	0.95
	ARq2	0.86		
	ARq3	0.86		
	ARq4	0.87		
	ARq5	0.88		
	ARq6	0.85		
Self-reflection and adaptation	ARq7	0.86	0.83	0.93
	ARq8	0.83		
	ARq9	0.86		
	ARq10	0.82		
	ARq11	0.76		
	ARq12	0.86		
Adaptive help-seeking	ARq13	0.87	0.86	0.92
	ARq14	0.84		
	ARq15	0.86		
	ARq16	0.87		
Negative affect and emotional response	ARq17	0.90	0.89	0.94
	ARq18	0.89		
	ARq19	0.92		
	ARq20	0.86		

#### Convergent validity

Convergent validity was confirmed with factor loadings (> 0.70) and Average Variance Extracted (AVE > 0.50; Fornell and Larcker, 1981; Collier, 2020). Loadings ranged from 0.66–0.92 (e.g., family support = 0.84, perseverance = 0.92). AVE values: PASS (0.75–0.78), AMS (0.70–0.83), ARS (0.83–0.89), all exceeding 0.50 (see Table 1).

#### Discriminant validity

Discriminant validity was established as AVE square roots exceeded inter-construct correlations (e.g., peer-teacher r = 0.352, shared variance = 0.124 < AVE = 0.76, 0.78; perseverance-affect r = 0.549 < AVE = 0.89, 0.83) (Collier, 2020). No correlations exceeded 0.85, confirming distinctiveness (see Table 2).

**Table 2:** Discriminant validity assessment.

**Discriminant validity assessment**							
**Perceived academic support**							
Correlation between constructs		1		2		3	
Perceived family support	1						
Perceived peer support		0.200^**^		1			
Perceived teacher support		0.185^**^		0.352^**^		1	
**Academic motivation**							
Correlation between constructs	1	2	3	4	5	6	7
1.Intrinsic motivation to know	1						
2.Intrinsic motivation toward the accomplishment	0.652^**^	1					
3.Intrinsic motivation to experience stimulation	0.569^**^	0.554^**^	1				
4.External regulation	0.270^**^	0.279^**^	0.321^**^	1			
5.Identified regulation	0.325^**^	0.292^**^	0.287^**^	0.404^**^	1		
6.Introjected regulation	0.430^**^	0.385^**^	0.402^**^	0.346^**^	0.456^**^	1	
7.Amotivation	−0.519^**^	−0.477^**^	−0.331^**^	−0.223^**^	−0.345^**^	−0.333^**^	1
**Academic resilience**							
Correlation between constructs	1	2	3	4			
1.Perseverance	1						
2.Self-reflection and adaptation	0.543^**^	1					
3.Adaptive help-seeking	0.584^**^	0.447^**^	1				
4.Negative affect and emotional response	0.549^**^	0.459^**^	0.493^**^	1			

^**^P < 0.001.

### Procedures of data collection and ethical considerations

#### Data collection procedures

Data collection involved two phases: a pilot study to validate adapted instruments and a main study for primary data. Instruments were first adapted to the local context, followed by a pilot test. The main study was conducted from July 24 to July 31, 2024. Questionnaires were administered in classrooms or separate rooms during students' free time to avoid disrupting academic schedules. The investigator and trained assistants provided guidance, with completion taking 25–30 min in both phases. Academic achievement data (GPA) were subsequently obtained from the university registrar.

#### Ethical considerations

Ethical approval was obtained from Bahir Dar University's College of Education Research Ethics Committee (Protocol No. 000723), adhering to institutional ethical standards. Participants were comprehensively informed about the study's objectives, methodology, procedures, and their rights to voluntarily participate, refuse, or withdraw without consequences. Verbal informed consent, aligned with university ethical protocols, was secured, ensuring full comprehension while prioritizing autonomy and confidentiality. Data privacy was guaranteed, with usage restricted to academic purposes, minimizing risks of unauthorized disclosure. This rigorous process enhanced transparency, cultivated trust, and promoted participant engagement, thereby strengthening data validity and reliability while upholding the highest standards of research integrity.

### Preliminary analysis

Before analysis, data quality was assessed to address potential biases from a cross-sectional design, such as poorly worded questions, social desirability, respondent carelessness, or encoding errors. Of 550 main study questionnaires, 47 (8.5%) were excluded due to incompleteness, leaving 503 (91.5% response rate) for analysis. Data were entered into SPSS-24 and IBM SPSS AMOS-23. Responses to perceived academic support, academic motivation, and academic resilience scales were recorded as provided, with negatively worded academic resilience items (2, 17, 18, 19) reverse-scored. Amotivation subscale items were not reversed, aligning with study objectives. The database was reviewed for accuracy using frequency distributions, confirming all scores ranged from 1 to 5 with no missing values. Minimum and maximum scores aligned with expectations.

### Statistical assumptions

Before to main data analysis, basic statistical assumptions univariate and multivariate outliers, normality, linearity, homoscedasticity, and multicollinearity were thoroughly assessed. Tukey's box plots showed no univariate outliers. Normality was confirmed with skewness (< 3) and kurtosis (< 10). Bivariate scatter plots indicated linear relationships. Mahalanobis distance values were below the critical threshold (20.515), indicating no multivariate outliers. Levene's test (*p* > 0.05) confirmed homoscedasticity. Tolerance and VIF statistics showed no multicollinearity issues. All assumptions were met, supporting the suitability of the data for analysis.

### Data analysis

This study examined the mediating roles of academic motivation and academic resilience in the relationship between perceived academic support and academic achievement. Two analytical approaches were employed:

#### Pearson product-moment correlation

This assessed associations among predictor variables (perceived academic support, academic motivation, academic resilience) and their strength of relationship with academic achievement, providing an initial overview of variable overlap.

#### Structural equation modeling (SEM)

SEM, conducted via AMOS-23, tested direct and indirect effects of perceived academic support on academic achievement through academic motivation and resilience, addressing the study's mediation hypotheses.

## Results

### Correlational analysis

A Pearson product-moment correlation analysis was conducted on all independent, mediators, and dependent variables. The results of the correlation analysis between perceived academic support, academic motivation, academic resilience, and academic achievement are presented in Table 3.

**Table 3:** Inter-correlations between the variables of the study.

**Variables**	**1**	**2**	**3**	**4**
1. Perceived academic support	1			
2. Academic motivation	0.324^**^	1		
3. Academic resilience	0.343^**^	0.552^**^	1	
4. Academic achievement	0.266^**^	0.454^**^	0.617^**^	1

^**^p < 0.001.

The results of the Pearson product-moment correlation coefficient presented in Table 3 indicate that perceived academic support has a significant and positive correlation with academic motivation (r =0.324, *p* < 0.001), academic resilience (r = 0.343, *p* < 0.001), and academic achievement (r = 0.266, *p* < 0.001). In addition, academic motivation is also significantly and positively correlated with both academic resilience (r = 0.552, *p* < 0.001) and academic achievement (r = 0.454, *p* < 0.001). Furthermore, academic resilience shows a significant and positive relationship with academic achievement (r = 0.617, *p* < 0.001).

### The serial mediating roles of academic motivation and academic resilience in the relationship between perceived academic support and academic achievement

**Figure 2:**
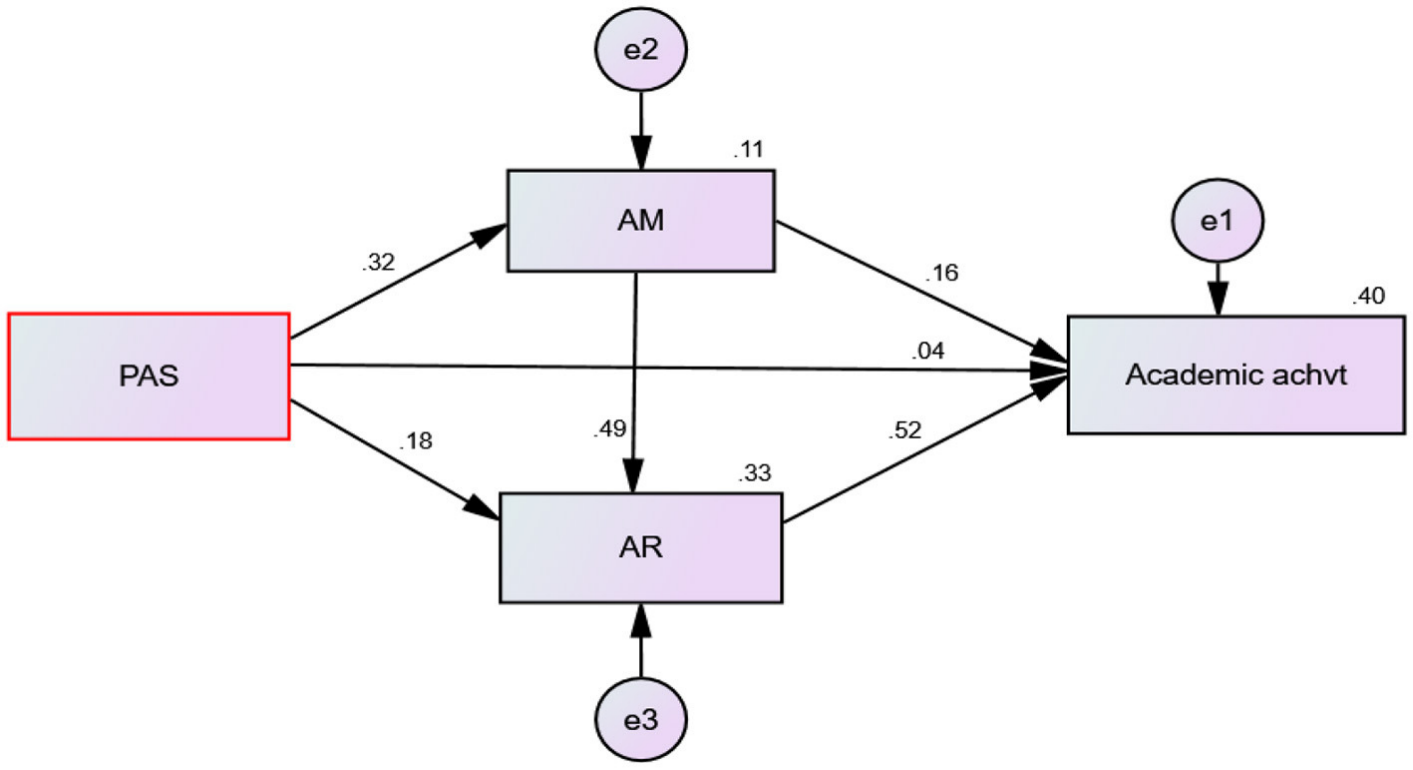
Path coefficients for predicting academic achievement from perceived academic support (PAS), academic motivation (AM), and academic resilience (AR) variables.

To explore the direct and indirect effects of students' perceived academic support on their academic achievement, a path analysis was conducted. As shown in Figure 2, the analysis is sought to investigate how academic motivation and resilience mediate the relationship between perceived academic support and academic achievement. The mediating roles of academic motivation and resilience in this relationship are discussed below.

To estimate the predictive power of the model, it is essential to examine the coefficient of determination (R^2^) for the endogenous variable. A higher R^2^ value indicates greater explanatory power and improved predictions of endogenous constructs (Hair et al., 2019). For example, an R^2^ value of 0.75 indicates substantial predictive power, whereas values of 0.50 and 0.25 suggest moderate and weak predictive power, respectively.

As shown in Figure 2, the coefficient of determination (R^2^) for academic achievement is 0.40, meaning that the combination of the three latent variables such as perceived academic support, academic motivation, and academic resilience accounted for 40% of the variance in academic achievement. In addition, the coefficient of determination (R^2^) for academic resilience is 0.33, which means that the 33% of the variance in academic resilience is explained by both perceived academic support and academic motivation. Moreover, the coefficient of determination (R^2^) for academic motivation is 0.11, meaning that perceived academic support explains 11% of the variance in academic motivation.

#### The direct effects of perceived academic support on academic achievement

As we have seen in Table 4, perceived academic support has a direct effect on the variables of academic motivation and academic resilience, but not on academic achievement. Besides, the variables of academic motivation and academic resilience have a direct effect on academic achievement. Moreover, academic motivation has a direct effect on academic resilience.

**Table 4:** The direct effect of perceived academic support on academic motivation, academic resilience, and academic achievement.

**Dependent variables**	**Path direction**	**Predictors**	**B estimate**	**β estimate**	**S.E**	**C.R**	***P***
Academic motivation	< -	PAS	0.507	0.324	0.066	7.680	^***^
Academic resilience	< -	PAS	0.293	0.183	0.061	4.764	^***^
Academic resilience	< -	AM	0.503	0.493	0.039	12.805	^***^
Academic achievement	< -	AM	0.006	0.155	0.002	3.687	^***^
Academic achievement	< -	AR	0.020	0.518	0.002	12.213	^***^
Academic achievement	< -	PAS	0.002	0.038	0.002	1.027	0.304

^***^p < 0.001.

PAS, perceived academic support; AM, academic motivation; AR, academic resilience.

The standardized beta coefficients (β) in this path model illustrate the strength and direction of relationships between variables in standardized units. As shown in Table 4, the analysis of the standardized path coefficients revealed that perceived academic support has a statistically significant relationship with academic motivation (β = 0.32, *p* < 0.001) and academic resilience (β = 0.18, *p* < 0.001), these suggesting that for every 1 standard deviation increase in perceived academic support leads to increase of 0.32, and 0.18 standard deviations in academic motivation and academic resilience respectively.

In addition, academic motivation is significantly related to both academic resilience (β = 0.49, *p* < 0.001) and academic achievement (β = 0.16, *p* < 0.001), indicating a 1 standard deviation increase in academic motivation leads to increases of 0.49 and 0.16 standard deviations in academic resilience and academic achievement, respectively. Moreover, academic resilience significantly related to academic achievement (β = 0.52, *p* < 0.001), indicating a 1 standard deviation increase in academic resilience leads to increases of 0.52 standard deviations in academic achievement. However, the direct causal association of perceived academic support and academic achievement (β = 0.038, *p* = 0.304) was not found to be statistically significant at a 0.05 alpha level.

#### The indirect and total effects of perceived academic support on academic achievement

The study aimed to explore the indirect and total effects of perceived academic support (the causal variable) on academic achievement, with a focus on how academic motivation and academic resilience mediate this relationship. To achieve this, the researcher utilized user-defined estimated in AMOS-23 to assess both the indirect and total effects of perceived academic support on academic achievement.

As summarized in Table 5, the specific indirect effects of perceived academic support on academic achievement, mediated by academic motivation, academic resilience, and through academic motivation via academic resilience (serial mediation), were significant, with *p*-values of 0.002, 0.001, and 0.002, respectively. This indicates that perceived academic support significantly influences academic achievement through these three pathways. Furthermore, the overall indirect effect (also known as the total indirect effect) of perceived academic support on academic achievement through these three variables was significant at p=0.001. Additionally, the combined total effect (both direct and indirect) of perceived academic support on academic achievement was also significant, with a *p*-value of 0.002. The table below details the indirect and total effects of perceived academic support on academic achievement via academic motivation, academic resilience, and the interaction between academic motivation and academic resilience.

**Table 5:** Indirect and total effects of perceived academic support on academic achievement via academic motivation, academic resilience, and academic motivation through academic resilience (user-defined results using AMOS).

**Parameters**		**Estimate**	**Lower bound**	**Upper bound**	***P***
*Specific indirect effect of:*	Mediators				
Perceived academic support	Via academic motivation	0.003	0.001	0.006	0.002
Perceived academic support	Via academic resilience	0.006	0.003	0.009	0.001
Perceived academic support	Via academic motivation through academic resilience	0.005	0.004	0.007	0.002
**Total indirect effect of:**					
Perceived academic support	Via academic motivation, via academic resilience, and academic motivation through academic resilience	0.014	0.011	0.018	0.001
**Total effect of:**					
Perceived academic support	(Direct effect + indirect effect)	0.017	0.010	0.022	0.002

The results presented in Table 5, indicate that all specific indirect effects, as well as the total indirect and total effects of perceived academic support on academic achievement, were statistically significant. This suggests that a better understanding of academic support enhances academic motivation, which in turn strengthens academic resilience, ultimately leading to improved academic achievement. Additionally, these findings highlight the critical role of perceived academic support in promoting both academic motivation and resilience, ultimately contributing to improved academic achievement.

However, the path analysis also revealed that the direct effects of perceived academic support on academic achievement were not significant, whereas, the indirect effect was significant, indicating that the relationship between perceived academic support and academic achievement is fully mediated by academic motivation and academic resilience.

## Discussion and implications

The study investigated the mediating roles of academic motivation and academic resilience in the relationship between perceived academic support and academic achievement among first-year university students, assessing both direct and indirect effects.

### Direct effect of perceived academic support on academic achievement

The analysis revealed that perceived academic support did not significantly predict academic achievement, consistent with prior studies (Tayfur and Ulupinar, 2016; Tinajero et al., 2020; Mackinnon, 2012; Nicpon et al., 2006). For instance, Mackinnon (2012) found total perceived support from family, peers, and academic sources insufficient to predict GPA, and Nicpon et al. (2006) noted negligible effects of social support on GPA. Plunkett et al. (2008) similarly reported that academic support from friends had minimal impact on academic success, with adolescents perceiving support from significant figures as less influential (Tinajero et al., 2020; Nicpon et al., 2006). This lack of direct effect may stem from sociocultural factors in Ethiopia, where collectivist norms prioritize communal over individual academic support, potentially diluting its direct impact on achievement (Hofstede, 2001). Additionally, students may rely more on intrinsic factors or informal support networks, such as extended family or community elders, not captured by standard measures (Yirdaw, 2016).

Conversely, some studies report positive correlations. Tinajero et al. (2020) found perceived support predictive of achievement, while De la Iglesia et al. (2014) noted higher social support, particularly among females, linked to better outcomes. Abdullah and Kong (2014) and Cheng et al. (2022) also identified positive relationships, especially with family support. These discrepancies may arise from methodological variations, differing support measures, or sample diversity.

### Mediating roles of academic motivation and resilience

The model confirmed significant indirect effects of perceived academic support on academic achievement through academic motivation and resilience. Higher perceived support enhanced motivation, which bolstered resilience, leading to improved academic performance. This aligns with research showing perceived support positively influences motivation (Kiefer et al., 2015; Reyes et al., 2022) and resilience (Fang et al., 2020; Prabhu and Shekhar, 2017). Students with elevated motivation (Yarin et al., 2022; Awofala et al., 2020; Vallerand et al., 1992) and resilience (Karabiyik, 2020; Yang and Wang, 2022) achieve better outcomes. Fang et al. (2020) further demonstrated resilience's full mediating role between support and achievement, supported by praise from families, teachers, and peers enhancing coping mechanisms (Sanders et al., 2016).

### Practical and policy implications

The findings of this study, which demonstrate that academic motivation and resilience fully mediate the relationship between perceived academic support and achievement among first-year university students, carry significant practical and policy implications for enhancing educational outcomes, particularly in the Ethiopian higher education context. The integration of Self-Determination Theory (SDT), Stress and Coping Theory, and Cassidy's resilience model underscores the pivotal role of motivation and resilience in transforming a non-significant direct effect of support into a significant indirect effect on achievement, offering actionable insights for institutional practices and policy development.

#### Practical implications

Universities can leverage these findings to implement structured interventions aimed at fostering academic motivation and resilience. Programs such as mentorship initiatives and goal-setting workshops, grounded in SDT's principles of autonomy, competence, and relatedness (Ryan and Deci, 2000), can enhance students' intrinsic motivation, thereby improving engagement and academic performance. Similarly, resilience-building interventions, such as peer support groups and stress management training, aligned with Stress and Coping Theory and Cassidy's resilience model, can equip students with adaptive coping strategies to navigate academic stressors, reducing attrition rates (Tamrat, 2021). Embedding these programs into first-year orientation and ongoing academic advising is critical to support the challenging transition to university, particularly in Ethiopia, where high dropout rates remain a pressing issue (Kelemu and Sabanci, 2018). These interventions should be tailored to the resource-constrained settings of institutions like Ethiopian Universities, utilizing cost-effective approaches such as peer-led initiatives to maximize impact.

#### Policy implications

At the institutional and national levels, policymakers should prioritize resource allocation for motivation and resilience-building programs within higher education frameworks. Integrating these evidence-based interventions into university policies, such as mandatory orientation programs or academic support services, can institutionalize support systems that address Ethiopia's educational challenges, including low retention and achievement rates. Furthermore, national education policies should encourage collaboration between universities and governmental bodies to scale these interventions across diverse Ethiopian institutions, ensuring equitable access to support. The study's findings also advocate for a broader policy shift toward student-centered learning environments, aligning with global educational psychology literature that emphasizes motivation and resilience as universal drivers of academic success.

By addressing both context-specific challenges in Ethiopia and contributing to global educational strategies, these practical and policy recommendations enhance the study's impact, offering a roadmap for universities and policymakers to foster student success through targeted, evidence-based interventions.

## Conclusions

In summary, this study highlights that correlation analysis revealed significant positive correlations among perceived academic support, academic motivation, academic resilience, and academic achievement. Moreover, the structural equation modeling (SEM) analysis found that while the direct effects of perceived academic support on academic achievement were not statistically significant, the indirect effects, mediated by academic motivation and academic resilience, were significant. This suggests that the relationship between perceived academic support and academic achievement is fully mediated by academic motivation and academic resilience. The results indicate that academic motivation and academic resilience are promising areas for intervention to enhance students' academic achievement.

## Limitations of the study and suggestions for future research

This study examined the mediating roles of academic motivation and resilience between perceived academic support and achievement among first-year university students. Key limitations include its cross-sectional design, which limits causal inference; a sample restricted to Injibara University with limited demographic diversity, reducing generalizability; reliance on self-report measures that may introduce bias; and the focus solely on university students. Future research should adopt longitudinal designs, include diverse samples from multiple institutions, utilize mixed-methods approaches for measurement, and explore these relationships across different educational levels to enhance validity and applicability.

## Data Availability

The raw data supporting the conclusions of this article will be made available by the authors, without undue reservation.
